# Novel High-Quality Sonographic Methods to Diagnose Muscle Wasting in Long-Stay Critically Ill Patients: Shear Wave Elastography, Superb Microvascular Imaging and Contrast-Enhanced Ultrasound

**DOI:** 10.3390/nu13072224

**Published:** 2021-06-29

**Authors:** Carmen Rosa Hernández-Socorro, Pedro Saavedra, Juan Carlos López-Fernández, Federico Lübbe-Vazquez, Sergio Ruiz-Santana

**Affiliations:** 1 Department of Radiology, Hospital Universitario de Gran Canaria Dr. Negrín, Universidad de Las Palmas de Gran Canaria, Barranco de la Ballena s/n, 35010 Las Palmas de Gran Canaria, Spain; 2 Department of Mathematics, Universidad de Las Palmas de Gran Canaria, 35010 Las Palmas de Gran Canaria, Spain; p.saavedra.santana@gmail.com; 3 Department of Neurology, Hospital Universitario de Gran Canaria Dr. Negrín, Universidad de Las Palmas de Gran Canaria, Barranco de la Ballena s/n, 35010 Las Palmas de Gran Canaria, Spain; jclopezfdez@gmail.com; 4 Department of Intensive Care, Hospital Universitario de Gran Canaria Dr. Negrín, Barranco de la Ballena s/n, 35010 Las Palmas de Gran Canaria, Spain; lubbe22@hotmail.com; 5 Department of Intensive Care, Hospital Universitario de Gran Canaria Dr. Negrín, Universidad de Las Palmas de Gran Canaria, Barranco de la Ballena s/n, 35010 Las Palmas de Gran Canaria, Spain; sruisan@gobiernodecanarias.org

**Keywords:** ultrasound, sarcopenia, quadriceps femoris, muscle weakness, muscle mass, critical illness, elastography

## Abstract

Novel ultrasound (US) methods are required to assess qualitative changes in the quadriceps rectus femoris (QRF) muscle when evaluating mechanically ventilated, long-stay ICU patients with suspected neuromuscular acquired weakness (ICUAW). Our aim was to analyze novel US muscle assessment methods in these patients versus healthy controls by carrying out a prospective observational study. Shear wave elastography (SWE) showed, with a receiver operating characteristic (ROC) curve of 0.972 (95% confidence interval (CI) = 0.916–1.000), that patients increased muscle stiffness associated with muscle fibrosis when diagnosed with ICUAW. We also performed, for the first time, superb microvascular imaging (SMI), which is an innovative US technique designed for imaging microvascularization unseen with color Doppler US, and observed that 53.8% of cases had significantly lower QRF muscle microvascular angiogenic activity than controls (*p* < 0.001). Finally, we used contrast-enhanced ultrasound (CEUS) to analyze maximum and minimum QRF muscle perfusion and obtained a ROC curve of 0.8, but when used as markers for SMI, their diagnostic capacity increased to 0.988 (CI = 0.965–1) and 0.932 (CI = 0.858–1), respectively. These findings show, for the first time, that these novel sonographic muscle methods should be used for their diagnostic capacity when assessing sarcopenic processes associated with this group of critically ill patients.

## 1. Introduction

An increasing number of ICU patients require long stays, lasting up to several months after surviving the first acute episode. A long ICU stay is usually defined as the requirement for mechanical ventilation and ICU therapy for more than 1 week and up to 3 weeks [1,2]. In addition, chronic critical illness (CCI) has been defined as an ICU length of stay of at least 8 days combined with at least one eligible diagnosis during hospitalization among the following: prolonged acute mechanical ventilation, sepsis, severe wounds, stroke and traumatic brain injury, and tracheotomy [3]. Secondary sarcopenia and its links to neuromuscular ICU-acquired weakness (ICUAW) in critically ill long-stay ICU patients have received far less research attention than their short-term counterparts.

We have recently described a new, reliable, structured ultrasound (US) protocol to assess secondary sarcopenia in long-stay ICU catabolic patients with ICUAW that allows clinicians to assess quantitative and qualitative changes in the quadriceps rectus femoris (QRF) muscle in severely ill, mechanically ventilated patients with clinically suspected ICUAW [4]. However, new bedside imaging diagnostic techniques to assess qualitative muscle wasting alterations in long-stay ICU patients or in CCI are needed because they are clinically useful secondary sarcopenic diagnostic tools.

Shear wave elastography usually relies on a focused acoustic beam generated by a US transducer that compresses the underlying tissue, thus inducing a local shear wave. The speed of that wave, also known as the shear wave elasticity (SWE), is then measured as it propagates through the tissue and is displayed as a parametric image or through a selective region of interest (ROI) analysis in meters per second or kilopascals (kPa), depending on whether measuring speed or elasticity in the US image. SWE provides a quantitative metric of tissue stiffness because it directly relates to the local shear elastic modulus. Therefore, the stiffer the tissue, the greater the SWE [5]. Muscle shear wave elastography is considered a high-quality measurement of muscle biomechanical properties [6]. However, although SWE muscle analysis has previously been used in musculoskeletal lesions [7,8], as far as we know, it has never been utilized in critically ill patients with ICUAW who were exposed to relevant muscular alterations to assess secondary sarcopenia.

Superb microvascular imaging (SMI) is an innovative US technique specifically designed for imaging very low-flow states, which uses a unique algorithm that allows for the visualization of diminutive vessels with slow velocity without using a sonographic contrast agent [9]. To date, although SMI has been used to detect blood flow signals in various conditions such as breast lesions [9], lymph nodes [10] or carcinomas [11], as far as we know, no clinical research with SMI technology for microvascular assessment has previously been reported in muscle blood flow studies in critically ill patients with neuromuscular secondary sarcopenia associated with ICUAW.

The use of intravascular contrast-enhanced ultrasound (CEUS) offers easily accessible visualization and quantification of skeletal muscle and the microcirculation of other tissues with almost no side effects. A previously described CEUS protocol was shown to be able to assess muscle microvascular flow [12]. CEUS has also been shown to be able to demonstrate well-delineated, circumscribed areas of impaired perfusion with hypoenhancement compared with surrounding muscle areas at the clinical level of a lesion in the arterial wash-in phase (0–30 s after intravenous administration). We propose that the use of intravascular CEUS may improve the ability of the US to assess muscle quality characteristics and distinguish between muscular abnormalities in critically ill patients and healthy controls. In addition, the use of intravascular CEUS may also enable the sonographic detection of other minor muscle injuries.

The aim of this study was to investigate novel, reliable methods to assess the quality of sonographically analyzed QRF muscle secondary sarcopenia in long-stay mechanically ventilated critically ill patients with suspected neuromuscular acquired weakness, without previous malnutrition at ICU admission, at the bedside [3]. To do so, in addition to previously described US standardized procedures [4], we performed QRF muscle shear wave elastography (SWE), superb microvascular imaging (SMI) and contrast-enhanced QRF muscle ultrasound (CEUS) studies and, particularly, their specific correlation with SMI for muscle microvascular evaluation analysis. We also collected the same data for matched healthy controls.

## 2. Materials and Methods

### 2.1. Patients and Healthy Controls

We conducted a prospective observational study in a 42-bed adult medical–surgical ICU at a tertiary university hospital in Las Palmas de Gran Canaria (Canary Islands, Spain) between July 2019 and December 2020. As previously stated, patients were not malnourished prior to ICU admission, needed prolonged mechanical ventilation and were expected to have an ICU stay longer than seven days. We defined prolonged mechanical ventilation as when the duration of mechanical ventilation was longer than 14 days [2].

In all of the studied patients, when neuromuscular acquired weakness was clinically suspected [13], the novel QRF muscle US tools were applied in addition to performing the previously published US examination protocol [4]. Clinical diagnosis of neuromuscular ICUAW was considered when the patient, once awake, presented with flaccid quadriparesis and hyporeflexia in the absence of other neurological, biochemical or central neurological damage [14], and had a diagnostic electromyogram (EMG) [15], a median Medical Research Council (MRC) score of less than four [16,17], or both. The electromyogram was performed in 17 of the patients, of which 15 met electrophysiological criteria for axonal polyneuropathy and the remaining two patients presented neurophysiological criteria for mixed axonal and demyelinating polyneuropathy. Likewise, we also conducted US QRF muscle assessments on age-, sex- and body mass index (BMI)-matched healthy controls. Patients who were not expected to survive longer than three days and those with primary neuromuscular pathology were excluded.

The following demographic and clinical data were obtained: age; sex; height; weight; BMI; Glasgow Coma Score (GCS), acute physiology, chronic health evaluation (Apache) II score and sequential organ failure assessment (SOFA) score at ICU admission; ICU admission and discharge date; and ICU admission diagnosis, length of stay (LOS) and the presence of sepsis. Additionally, we collected data on the following organ failures, also upon ICU admission: respiratory, cardiovascular, renal, hepatic, hematologic and gastrointestinal. Finally, corticosteroid treatment and neuromuscular blocking treatment data were also collected.

### 2.2. Novel High-Quality Quadriceps Rectus Femoris US Methods for Sarcopenic Assessment

We performed the US assessment with an Aplio 500 US device (Canon Medical Systems Corporation, Tokyo, Japan), with 10–12 MHz small parts and a multifrequency linear-array probe (width of probe: 38–58 mm), on all patients for whom a neuromuscular acquired weakness diagnosis was considered appropriate, as well as on the healthy controls. During the assessment, the participants lay in the supine position with their arms supinated and their knees relaxed and fully extended.

The probe was coated with a suitable water-soluble transmission gel to provide acoustic contact without depression of the dermal surface, and it was aligned perpendicularly to the longitudinal and transversal axes of the QRF muscles with the aim of obtaining transverse and longitudinal images. To obtain the most accurate data of each QRF muscle for patients and controls, we performed at least three longitudinal and three transversal images on each QRF muscle and in B-Mode, M-Mode, Doppler, SMI and SWE. Regarding CEUS images, this contrast was given once on each leg and obtained at least three images on each QRF muscle. Once we collected all the images, we analyzed the numeric data on each one of them, and as a result, we calculated the median value of each data gathering. Image files were stored on the US device computer. Since muscle dimensions change with contraction and/or relaxation and the studied muscle is more compressible in a relaxed state [18,19,20], the assessment was performed without compression. The acquisition site was located two-thirds of the way along the femur length, measured between the upper pole of the patella and the anterior superior iliac spine. We measured the exact site with electronic calipers so that once the muscle was imaged, its boundaries could be identified and measured. For greater accuracy, averaged bilateral measurements were estimated. All sonographic exams were performed by a single examiner.

In all of the performed studies, we first used real-time B-mode US scanning to assess muscle quantity. Therefore, we measured the cross-sectional area (CSA) in cm^2^ and muscle thickness in mm and explored for the presence of edema in the subcutaneous tissue and for intramuscular and interfacial fluid. Muscle quality was first assessed in four categories according to its echogenicity by using a specifically designed scale, previously protocolized by us [4]. The scale was as follows: homogenous hypoechogenicity (Category 1); heterogeneous hypoechogenicity (Category 2); fat infiltration (Category 3); muscle fasciitis and/or necrosis (Category4) [4]. We also used conventional color Doppler US to assess QRF vascularization and, therefore, the angiogenic muscle activity. Additionally, we use M-mode US to demonstrate the presence or absence of fasciculations because it captures the mechanical event of the muscle contraction.

#### 2.2.1. Shear Wave Ultrasound Elastography (SWE)

During SWE image acquisition, the transducer was coated with a suitable water-soluble transmission gel to provide acoustic contact without depression of the dermal surface, and it was aligned perpendicularly to the longitudinal and transversal axes of the QRF muscles in order to achieve accurate and reliable SWE measurements. The position of the patient, muscle contraction and the pressure applied to the muscle by the probe can influence the SWE value. To limit bias, in addition to the afore mentioned amount of gel, we were careful not to apply any pressure to the muscle during the evaluation. The SWE data from each patient was obtained by using Aplio 500 US device, which has a specific software named: 2D Shear Wave. The probe must be perpendicular to the muscle to be able to measure the SWE. Then, we will obtain an image with two screens: on the left side, it showed the elastography (kPa) or speed (m/s) and on the right side, the propagation map (arrival time contour). To have a reliable measure of the propagation map, all lines should be smooth (not necessarily straight) and parallel to each other. If the lines are in a zigzag and not parallel to each other is an unreliable measure. Regarding the Region of interest (ROI), it is a circle with 10 mm of diameter and to obtain a valid ROI, and we must place it where the propagation map lines are parallel to each other and smooth. We measured for each longitudinal or transversal scan at least three or four ROIs. Afterwards, according to the obtained measures, we calculated the median values. ROI values were calculated as kPa, and the 2DSWE was color-coded in dark blue (less than 36 kPa), light blue (36–72 kPa) and green, yellow and red (greater than 180 kPa) [21].

**Figure 1:**
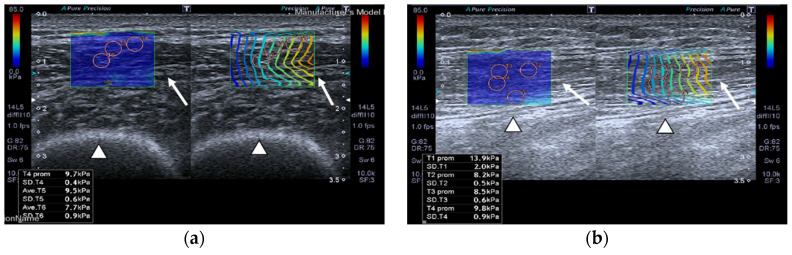

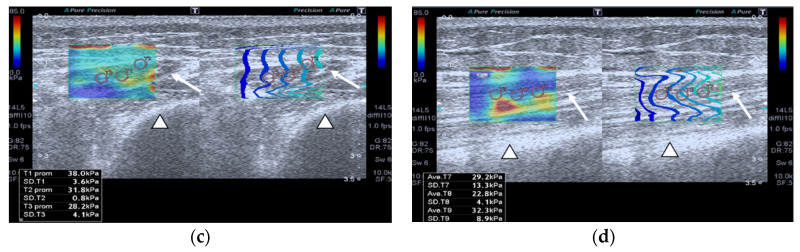
Quadriceps rectus femoris (QRF) muscle ultrasound (US) scan and shear wave elastography (SWE) with several regions of interest (ROIs) (pink circles) measured across the muscle of a matched healthy control (63 years old). SWE shows high muscle elasticity. (**a**) Transversal QRF muscle (arrow) US scan, femur (arrowhead); (**b**) longitudinal QRF muscle (arrow) US scan, femur (arrowhead). Patient with multiorgan failure (67 years old). SWE shows muscle stiffness with several regions of interest (ROIs) (pink circles). (**c**) Transversal QRF muscle (arrow) US scan, femur (arrowhead); (**d**) longitudinal QRF muscle (arrow) US scan, femur (arrowhead).

SWE images of a case and a healthy control are shown in Figure 1.

#### 2.2.2. Superb Microvascular Imaging (SMI)

SMI was performed to observe and record the vascular structures of the QRF muscle. The following parameters were set for the SMI examination: color velocity scale = 1 to 2 cm/s; color frequency = 5–7 MHz; color frequency frame rate > 30 frames per second; the gain setting was adjusted to show optimal vascular imaging information. Both color SMI (cSMI) and monochrome SMI (mSMI) were used in all subjects, but only the mSMI was used to assess the muscle vascularity in this study due to its higher sensitivity.

Therefore, SMI was performed in monochromatic mode, and the SMI settings were standardized to the manufacturer’s recommendations of a low-velocity range (<2 cm/s) and high frame rate with minimal flash artifacts. The monochromatic mode was chosen, as stated above, due to its high sensitivity for low-and slow-velocity blood flow detection. We evaluated two kinds of parameters, quantitative and qualitative. With reference to the quantitative, it is analyzed by the vascular index, which is shown in percentage (%). This parameter shows the ratio between the Doppler signal pixel and the data obtained from the studied muscle. It can be calculated by the software VI test app of the Aplio 500 US device. On the other hand, we have also studied the qualitative parameters of the muscle. Those are: vessel morphology, vessel distribution and the presence of penetrating vessels.

- Vessel morphology can be categorized as simple, which manifests as dot-like or linear forms or complex, which can be found as branching or shunting types.
- Vessel distribution can be classified as peripheral, which shows with all vessels located at the margin; or central, which displays with any vessel that can be detected within the studied muscle.
- Presence of penetrating vessels, which is shown as a vessel with high vascularization.

Still images from the SMI of the target QRF muscle were obtained and archived on a picture archiving and communication system. All obtained data were shown as a percentage of the microvascularization presence or absence. The additional time required for the SMI analysis was usually less than 10 s for most patients.

**Figure 2:**
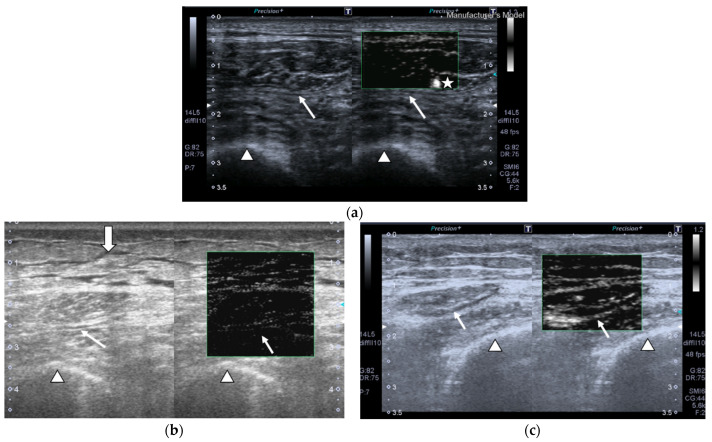
Transversal QRF muscle US scan and the superb microvascular imaging (SMI) built-in software that indicates microvascularization. (**a**)Transversal QRF muscle (arrow) US scan, femur (arrowhead). SMI image shows dot-like vessels and a penetrating vessel (star) for a matched healthy control (60 years old). (**b**) Transversal QRF muscle (arrow) US scan, femur (arrowhead); subcutaneous edema with intramuscular and interfacial fluid (thick arrow). SMI image shows dot-like vessels for a patient with multiorgan failure (58 years old). (**c**) Transversal QRF muscle (arrow) US scan, femur (arrowhead). SMI image shows minimal linear vessels for another patient with multiorgan failure (51 years old).

SMI images of a patient and a healthy control are displayed in Figure 2.

#### 2.2.3. Contrast-Enhanced Ultrasound (CEUS)

**Figure 3:**
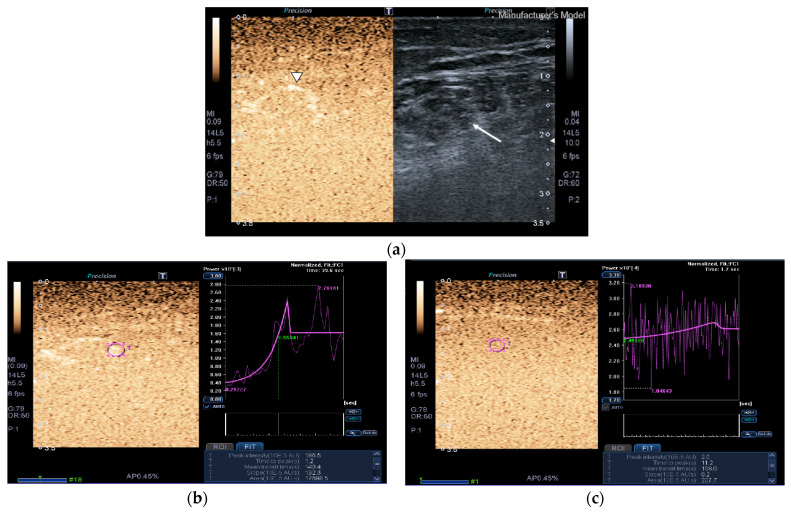
Transversal QRF muscle of a matched healthy control (85 years old). (**a**) Transversal ultrasonography scan shows the QRF muscle (arrow) and vascularization after contrast-enhanced ultrasound (CEUS) administration (arrowhead). CEUS image and time–intensity curve analysis are shown using the built-in software. (**b**) ROI (pink circle) in the area of strongest enhancement indicates maximum perfusion in the QRF. (**c**) ROI (pink circle) in the area of lowest enhancement indicates minimum perfusion in the QRF.

Both patients and healthy controls underwent a CEUS assessment of QRF muscle blood flow. An intravenous bolus injection of 4.8 mL of SF_6_ (sulfur hexafluoride) microbubbles (Sono-Vue^®^, Bracco, Italy), an intravascular contrast agent, was given via a cubital intravenous line. The microbubbles are recovered and stabilized by a phospholipid membrane. Additionally, this contrast is purely intravascular, meaning that the microbubbles will not go through the endothelium. Moreover, the size of the microbubbles is smaller than that of red blood cells. The contrast agent is eliminated from the body via expired air and has few side effects, which can include headache and abdominal pain. It can be used in patients that suffer from renal failure but cannot be used in patients with recent acute coronary syndrome or clinically unstable ischemic cardiac disease. CEUS can be measured quantitatively and qualitatively. In fact, the CEUS enhancement pattern (ROI) is a qualitative parameter and is shown in Figure 3a. Regarding the quantitative parameter, it is measured by a time–intensity curve that is obtained with our built-in software from Aplio 500 US device. After the infusion, the distribution of the contrast agent was visualized in the early arterial phase, which allowed us to assess the microvascular flow of the QRF muscle [12]. CEUS images were acquired, and time–intensity curve analysis of a CEUS video clip was then performed. After setting an ROI (pink circle) in the area of strongest enhancement, the following quantitative parameters were automatically calculated: peak intensity(×10^−5^ arbitrary units [AU]), which is the maximum intensity of the time–intensity curve; time to peak (seconds), the time needed to reach the peak intensity; mean transit time(seconds), the time when the intensity is higher than the mean value; slope(×10^−5^ AU/seconds), which is the maximum wash-in velocity of the contrast agent; and area under the curve(×10^−5^ AU · seconds), which is the integral value of the curve was associated with the total blood volume and the sum of the wash-in area and wash-out area.

**Figure 4:**
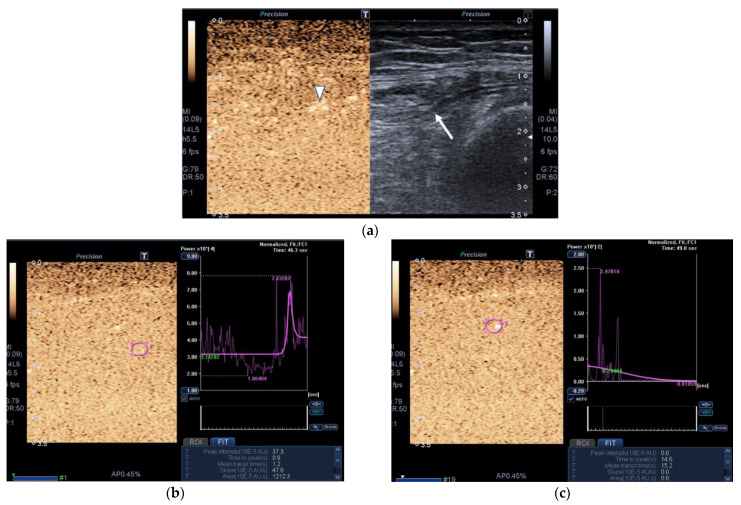
Transversal QRF muscle of a patient with multiorgan failure (77 years old). (**a**). Transversal ultrasonography scan shows the QRF muscle (arrow) and vascularization after contrast-enhanced ultrasound (CEUS) administration (arrowhead). CEUS imaging and time–intensity curve analysis are shown using the built-in software. (**b**) ROI (pink circle) in the area of strongest enhancement indicates maximum perfusion in the QRF. (**c**) ROI (pink circle) in the area of lowest enhancement indicates minimum perfusion in the QRF.

Images of the peak maximum and minimum CEUS intensity of a patient and a healthy control are shown in Figure 3 and Figure 4.

The hospital institutional review board approved the study (protocol number: 2019-344-1, 25 July 2019). Written informed consent was obtained from patients or close relatives.

### 2.3. Statistical Analysis

Categorical variables were expressed as frequencies and percentages, and continuous variables were expressed as means and standard deviations (SD) when data followed a normal distribution or as medians and interquartile ranges (IQR = 25th–75th percentile) when the distribution departed from normality. The percentages were compared, as appropriate, using the chi-square (χ^2^) test or the exact Fisher test; the means were compared by a *t*-test, and the medians were compared using the Wilcoxon test for independent data. A receiver operating characteristic (ROC) analysis was conducted to determine the discriminant power of the muscle area for the outcome. The area under the corresponding ROC curve was estimated using the means of 95% confidence intervals. The discriminant threshold (corner closest to (0, 1)) was chosen as that which minimized the function:

(1 − Sensitivity)^2^ + (1 − Specificity)^2^

For the obtained predictor, the sensitivity and specificity were estimated using the means of 95% confidence intervals. Statistical significance was set at *p* < 0.05. Data were analyzed using the R package, version 3.6.1 (R Development Core Team, 2019).

## 3. Results

During the study period, 1746 patients were admitted to the ICU, of which 960 were mechanically ventilated and 362 were ventilated for longer than 7 days; eventually, 167 of these patients had prolonged mechanical ventilation. Neuromuscular acquired weakness was clinically suspected in 26 out of 167 patients (15.5%), to whom the novel QRF-US methods and the study protocol were applied. The median time on mechanical ventilation was 51 days (IQR: 34.2–92.5). The variables for the entire cohort and for each group are summarized in Table 1. There were no significant differences in grouping based on demographics.

The clinical characteristics of the patients are shown in Table 2. The patients were critically ill upon ICU admission, as shown by the studied severity scores. Most of them (76.4%) had a multiorgan failure (particularly respiratory, renal and cardiovascular failure). Additionally, 84.6% of them were septic upon ICU admission, and 61.5% and 34.6% received corticosteroids or neuromuscular blockers during their ICU stay, respectively. The median time between ICU admission and the clinical suspicion of ICUAW/performance of the QRF muscle ultrasound study was 32 days.

As displayed in Table 1, the median SWE values in kilopascals were significantly greater in the patients compared to the control group (*p* < 0.001). It showed an area under the ROC curve of 0.972 (95% CI = 0.916–1.000). Additionally, 53.8% of the patients had significantly lower QRF muscle microvascular angiogenic activity levels, as it was detected by SMI if we compared those to the controls, which all had normal, hundred percent, microvascularization (*p* < 0.001). Measurements of the maximum and minimum QRF muscle perfusion, as assessed by CEUS, were both significantly lower in the patients versus the controls (*p* < 0.001). Their diagnostic value was shown in the areas under the ROC curves (0.801(95% CI = 0.668–0.934) and 0.817 (95% CI = 0.682–0.951), respectively). Measurements of minimum and maximum CEUS, as markers of SMI, both had an area under the receiver operating characteristic (ROC) curve over 0.93 and 0.98, respectively, as shown in Table 3.

Additionally, as shown in Table 1, the QRF muscle area and thickness significantly decreased (*p* < 0.001) in the patient group compared to the control group. Their diagnostic values had an area under the ROC curve of 0.971 (95% CI = 0.932–1.000) and 0.950 (95% CI = 0.893–1.000), respectively. Significantly greater levels of intramuscular/interfacial fluid and subcutaneous edema (*p* < 0.001) were seen in the patients (80.8% and 65.3%, respectively) compared to 0% for both factors in the control group. Echogenicity was also significantly different in the patients versus controls (*p* < 0.001). None of the cases were graded as Categories 1 or 2, but 42.3% had fat infiltration, and 57.7% had muscle necrosis and fasciitis and were thus graded as Categories 3 and 4, respectively. In addition, 69.2% of patients had significantly lower numbers of detected fasciculations on the muscle if we compared them to the healthy controls, who all had 100% of fasciculations (*p* < 0.001). Of note, there was no significant difference in subcutaneous tissue thickness between both study groups (*p* = 1). We also visualized QRF muscle vascularization with color Doppler US, which allowed us to assess blood flow changes in the studied subjects; significantly lower QRF muscle angiogenic activity was observed in 53.8% of the patients compared to the healthy controls, who had normal vascularization (*p* < 0.001). ROC curve analysis results for all of the studied QRF muscle wasting markers are shown in Table 4.

## 4. Discussion

We prospectively studied novel high-quality US methods in an important group of critically ill patients at risk of prolonged ICU and hospital stay or death, along with increased use of health resources. Most of the studied patients were septic, had a multiorgan failure and received corticosteroids or neuromuscular blocking agents, which are well-recognized risk factors for ICUAW [14,15]. Due to these factors, they were at risk of developing secondary sarcopenia, which eventually leads to musculoskeletal weakness and physical damage that can persist for years in those who survive a prolonged ICU stay.

On applying the researched novel high-quality US tools in these patients, we found that QRF muscle SWE showed a significant increase in muscle stiffness, measured in kPa, in patients compared to the control group (*p* < 0.001), with an outstanding area under the ROC curve of 0.97. This finding suggests that the QRF muscles in these studied patients became stiffer, indicating a significant increase in muscle fibrosis. We also found that 53.8% of the patients had significantly lower QRF muscle microvascular angiogenic activity, as detected by SMI, than the controls, who had normal microvascularization (*p* < 0.001). As far as we know, this is the first time that these techniques have been applied to this category of critically ill patients. This demonstrates that we are able to detect changes in muscle stiffness and low flow microvascularization with this technique that cannot be visualized using the regular color Doppler US technique. Additionally, maximum and minimum QRF muscle perfusion levels, as assessed by CEUS, were significantly lower in patients than in the controls (*p* < 0.001). This finding indicates less muscle perfusion and, consequently, a loss of their muscle biomechanical properties. However, muscle perfusion persistence detection is also a good preemptive indicator of muscle strength and functional recovery.

When we applied our usual muscle US protocol, we found that QRF muscle area and thickness significantly decreased in the studied patients compared to the control group (*p* < 0.001), confirming our and others’ previous findings of US-diagnosed muscle quantity loss during an ICU stay [4,22]. Interestingly, QRF cross-sectional area has also been found to be a more reliable proxy for muscle strength in an ICU setting, where volitional and non-volitional muscle strength measurements are challenging [23]. As expected, significantly greater levels of subcutaneous edema and intramuscular/interfacial fluid (*p* < 0.001) were also seen in the patients, compared with the absence of these factors in the controls, which demonstrates that the US can easily detect fluid displacement in these patients.

Echogenicity, another relevant muscle quality characteristic in US, was also significantly different in patients versus controls (*p* < 0.001). Even though during severe catabolic illness, normal musculoskeletal tissue is slowly replaced by fibrous or fat tissues (thus progressively increasing its echogenicity), we observed that none of the cases were graded as Categories 1 and 2, most probably due to the patients having a prolonged ICU stay and, therefore, only exhibiting a greater degree of fat infiltration (Category 3) or muscle necrosis and fasciitis (Category 4). In addition, we also observed that 69.2% of patients had significantly lower numbers of detected fasciculations compared to 100% of healthy controls (*p* < 0.001). These findings are similar to those obtained in our previous study [4], and they may also be explained by the fact that the patients presented with muscle-specific myofiber alterations [24]. The color Doppler US showed significantly lower QRF muscle angiogenic activity in 53.8% of the patients compared to the healthy controls, who had normal vascularization (*p* < 0.001), indicating a decrease in muscle angiogenesis, which also confirms previous findings [4].

Regarding methods for musculoskeletal evaluation in critically ill patients, attention has mainly been focused on computed tomography (CT), bioelectrical impedance spectroscopy (BIS) and US [25]. CT allows for muscle quantity and quality assessment but requires patient radiation and, most frequently, moving the patient to radiology department facilities. However, it has a relevant prognostic value, and it has been demonstrated that low skeletal muscle quality at ICU admission (as assessed by CT-derived skeletal muscle density) is independently associated with higher 6-month mortality in mechanically ventilated patients, thus reinforcing the importance of muscle quality and quantity as prognostic factors in the ICU [26]. Serial BIS [27] may be less accessible at the bedside than US but also requires less training for its use. However, it may be misleading, as its measurements are mainly linked to inaccuracies due to large fluxes in fluid status in critically ill patients. This fact, coupled with the lack of reliable weight measures in critical care, the lack of predictive equations for this cohort, and the limitations in positioning the patient for accurate measurements, reduces its usefulness [6]; there is still a need for more research in this specific area. US measurements, however, have demonstrated utility in measuring declines in muscle mass and quality in critically ill patients [2,4,28]. US assessment of musculoskeletal composition by applying the described novel US techniques combined with those previously protocolized by our team provides a unique opportunity to develop improved methods of secondary sarcopenia diagnosis and potential recovery when ICUAW is suspected, based on objective data.

Concerning SWE, although there has been an increase in the number of studies regarding musculoskeletal elastography [7], this technique has barely been studied in the setting of critically ill patients [29], particularly in those patients with suspected ICUAW and on long-term mechanical ventilation. SWE, as stated earlier, is a method of US imaging based on the detection of shear wave propagation through tissues. By using inversion algorithms, this method maps the waves into elastograms and determines the stiffness of the tissue by measuring the shear modulus value [30,31]. SWE gives a spatial representation of soft tissue stiffness and provides measures of muscle quality, and it seems to be a reliable technique to evaluate limb muscles and the diaphragm in both critically ill patients and healthy controls [29]. SWE has also been established as an excellent diagnostic method for the fibrosis stage, both in nonalcoholic fatty liver disease [32] and in several other non-hepatic applications [7]. In skeletal muscles, it provides a two-dimensional representation and quantifiable measurement of their mechanical properties and an estimate of muscle fibrosis [21]. Although it has been shown that SWE muscle analysis may provide new data about muscle quality during critical illness [29], as far as we know, it has not previously been evaluated in long-stay critically ill patients with suspected ICUAW that are exposed to relevant muscular alterations.

In this study, we found, when measuring QRF muscle elasticity, that median SWE values (in kilopascals) were significantly greater in patients compared to the healthy control group. It is important to stress that SWE values, but not echogenicity, are associated with muscle fibrosis and that high shear modulus values have been associated with muscle stiffness, while low shear modulus values have been linked to atrophy in chronic myopathies [29]. Therefore, our results, with an outstanding area under the ROC curve, confirm these findings and show that our studied patients developed significant rigidness of the muscle, which is associated with muscle fibrosis when first diagnosed with ICUAW in long-term mechanically ventilated critically ill patients.

We performed SMI to depict the vascular structures of the QRF muscle. It is an innovative software US technique specifically designed for imaging very low flow states, which uses a unique algorithm that allows for the visualization of diminutive vessels at slow velocity without using a contrast agent [9]. To date, although SMI has been used to detect blood flow signals in various serious clinical conditions (mainly in breast lesions [9,10,11]), as far as we know, no clinical research using SMI has been reported in muscle blood flow studies in critically ill patients with neuromuscular damage due to secondary sarcopenia associated with ICUAW. We found that around half of the studied patients had significantly lower QRF muscle microvascular angiogenic activity, as detected by SMI, than the control patients, who had normal vascularization. As far as we know, this is the first time that this technique has been applied to such long-term mechanically ventilated critically ill patients. This means that using this technique makes it possible to detect tiny changes in muscle flow microvascularization that cannot be visualized by color Doppler US alone. It is important to be able to detect this QRF muscle low flow because it most likely has muscle recovery prognostic importance, which should be confirmed in future studies.

CEUS may be the preferred method for the assessment of defective skeletal muscle blood flow responses to exercise and for investigating and quantifying responses to therapy [33]. CEUS is a non-invasive sonographic technique for quantitative imaging that can be used to assess muscle vascular perfusion. For this technique, a contrast agent containing inert gas-filled microbubbles, which are smaller than red blood cells in size, is injected into the bloodstream. Once the microbubbles are exposed to the high-energy ultrasound in the region of interest (ROI), they are destroyed. The destroyed microbubbles are replenished by the neighboring blood vessels, and afterwards, the microbubble intensity is gradually restored in the ROI. The kinetics of the microbubbles in the ROI are used to estimate perfusion indices, as previously published; the concentration of microbubbles when fully replenished is proportional to the microvascular blood volume (MBV), and the rate at which the microbubbles replenish determines the microvascular flow velocity (MFV). MBV represents the total amount of capillaries participating in the microcirculation at a given moment, whereas blood flow (MBF) is the product of blood volume and flow velocity [33].

In our study, the patients and healthy controls underwent CEUS assessment of QRF muscle blood flow. Maximum and minimum QRF muscle perfusion levels, as assessed by CEUS, showed significantly lower values in patients compared to controls (*p* < 0.001). This finding indicates lesser QRF muscle perfusion and a decrease in angiogenic activity, which would entail a loss of their biomechanical properties. CEUS by itself also contributes to the analysis of QRF muscle perfusion, but its excellent diagnostic capacity, as shown by an area under the ROC curve of 0.8, is slightly lower than that of other more relevant QRF muscle biomarker tools researched in this study, such as SMI. However, when we use CEUS maximum and minimum values as markers of SMI, they show a better sensitivity and specificity, obtaining a ROC curve value of 0.93 for CEUS minimum and 0.98 for CEUS maximum. As far as we know, this is the first time that this technique has been used to diagnose QRF muscle perfusion alterations in long-term mechanically ventilated critically ill patients. We believe that with these two novel qualitative methods, one can not only establish the patient’s current angiogenic activity, but it would also be feasible to forecast, in upcoming additional studies, the patient’s recovery prognosis in the long term.

## 5. Conclusions

In this study, we were able to assess specific qualitative changes in the QRF muscle by applying three novel US methods to mechanically ventilated long-stay ICU patients with clinically suspected neuromuscular acquired weakness. Among the newly studied US tools, SWE showed, with an outstanding area under the ROC curve, that the studied patients developed serious muscle rigidness associated with muscle fibrosis when first diagnosed with ICUAW, which has relevant diagnostic and prognostic consequences. We also performed SMI in these patients for the first time; SMI is a new US software analysis that allowed us to detect tiny changes in muscle flow microvascularization unseen with color Doppler US. Being able to detect low muscle flow allows to demonstrate muscle viability, and it has recovery prognostic importance. Finally, we used CEUS to analyze QRF muscle perfusion; despite its excellent diagnostic capacity, as shown by an area under the ROC curve value of 0.8, it performed slightly poorer than the other QRF muscle biomarker tools researched in this study, but when used as a marker of SMI, its diagnostic capacity increased to over 0.9 in terms of area under the ROC curve value. These findings are relevant because they show, for the first time, that these novel sonographic muscle tools can be used to assess the muscle quantity and quality wasting process in this specific group of critically ill patients and should, due to their clinical relevance, be added to sonographic musculoskeletal diagnostic protocols.

## Figures and Tables

**Table 1:** Characteristics of the cases and controls.

	Overall N = 43	Controls N = 17	Cases N = 26	*p*-Value
Age (years)	64.8 ± 14.0	61.5 ± 19.6	67.0 ± 8.4	0.213
Age ≥ 65 years	22 (51.2)	7 (41.2)	15 (57.7)	0.289
Sex				0.234
Male	25 (58.1)	8 (47.1)	17 (65.4)	
Female	18 (41.9)	9 (52.9)	9 (34.6)	
Body mass index (kg/m^2^)	27.7 ± 8.2	26.5 ± 5.6	28.5 ± 9.6	0.438
SWE (kPa)	24.9 (11.7–30.1)	11.6 (10.6–12.0)	28.2 (26.0–32.0)	<0.001
SMI	31 (72.0)	17 (100.0)	14 (53.8)	<0.001
Maximum CEUS	290 (49–1277)	553 (299–7776)	92 (25–609)	0.001
Minimum CEUS	14.7 (0.5–161.8)	52.5 (39.4–207.7)	1.5 (0.0–13.9)	<0.001
Muscle area (cm^2^)	2.3 (1.4–3.5)	3.8 (3.4–4.2)	1.5 (1.2–1.8)	<0.001
Muscle thickness (mm)	8.0 (5.8–10.3)	11.4 (9.8–13.1)	6.3 (4.7–7.5)	<0.001
Intermuscular/interfacial fluid	21 (48.8)	0	21 (80.8)	<0.001
Subcutaneous edema	17 (39.5)	0	17 (65.3)	<0.001
Echogenicity				<0.001
1	17 (39.5)	17 (100.0)	0	
3	11 (25.6)	0	11 (42.3)	
4	15 (34.9)	0	15 (57.7)	
Fasciculations	35 (81.3)	17 (100.0)	18 (69.2)	<0.001
Subcutaneous tissue thickness (mm)	10.1 (8.2–13.5)	10.4 (8.2–13.0)	9.8 (8.3–13.5)	1
Doppler	31 (72.0)	17 (100.0)	14 (53.8)	<0.001

Data are means ± SD, frequencies (%) and medians (IQR). Abbreviations: SD = standard deviation; IQR = interquartile range; SWE = shear wave elastography; kPa = kilopascals; SMI = superb microvascular imaging; CEUS = contrast-enhanced ultrasound.

**Table 2:** Patients’ clinical characteristics.

Apache-II, mean ± SD	19.9 ± 6.6
Sofa score at ICU admission, median (IQR)	7 (6–9.8)
GCS at ICU admission, median (IQR)	15 (11.5–15)
Time between ICU admission and QRF-US in days, median (IQR)	32 (18–46.2)
Sepsis at ICU admission, n (%)	22 (84.6)
Failures, n (%)	
Multiorgan	20 (76.4)
Respiratory	20 (76.9)
Cardiovascular	10 (38.5)
Renal, n (%)	18 (69.2)
Hepatic	4 (15.4)
Hematological	1 (3.9)
Gastrointestinal	1 (3.9)
Corticosteroids, n (%)	16 (61.5)
Neuromuscular blocking agents, n (%)	9 (34.6)
ICU days, median (IQR)	64 (39.8–106)
Hospital days, median (IQR)	103 (55.8–142.2)

Data are means ± SD, frequencies (%) and medians (IQR). Abbreviations: ICU = intensive care unit; GCS = Glasgow Coma Scale; QRF-US: quadriceps rectus femoris ultrasonogram.

**Table 3:** CEUS as a marker of SMI.

	AUC (95% CI)	Optimal Threshold *	Sensitivity (95% CI)	Specificity (95% CI)
Minimum CEUS	0.932 (0.858–1)	1.65	85.7 (57.2–98.2)	93.1 (77.2–99.2)
Maximum CEUS	0.988 (0.965–1)	119	92.9 (66.1–99.8)	96.6 (82.2–99.9)

Abbreviations: CEUS = contrast-enhanced ultrasound; SMI = superb microvascular imaging; AUC = area under the curve; (*) Point closest-to-(0,1) corner.

**Table 4:** Diagnosis values of the QRF muscle markers.

	AUC	Threshold *	Sensitivity (%)	Specificity (*)
SWE (kPa)	0.972 (0.916–1.000)	18.70	96.0 (79.6–99.9)	100.0 (80.5–100.0)
Maximum CEUS	0.801 (0.668–0.934)	251.5	69.2 (48.2–85.7)	94.1 (71.3–99.9)
Minimum CEUS	0.817 (0.682–0.951)	17.1	76.9 (56.4–91.0)	88.2 (63.6–98.5)
Muscle area	0.971 (0.932–1.000)	2.79	92.3 (74.9–99.1)	88.2 (63.6–98.5)
Muscle thickness	0.950 (0.893 – 1.000)	8.20	80.8 (60.6–93.4)	88.2 (63.6–98.5)
Subcutaneous tissue thickness	0.500 (0.321–0.679)	10.35	61.5 (40.6–79.8)	52.9 (27.8–77.0)

All estimations are shown with the 95% CI. Abbreviations: QRF = quadriceps rectus femoris; AUC = area under the curve; SWE: shear wave elastography; kPa: kilopascals; CEUS: contrast-enhanced ultrasound. (*) Point closest-to-(0,1) corner.

## Data Availability

Data are available on request due to institutional restrictions.
